# Differential Effects of Iron Chelates vs. Iron Salts on Induction of Pro-Oncogenic Amphiregulin and Pro-Inflammatory COX-2 in Human Intestinal Adenocarcinoma Cell Lines

**DOI:** 10.3390/ijms24065507

**Published:** 2023-03-14

**Authors:** Agata Tarczykowska, Niklas Engström, Darja Dobermann, Jonathan Powell, Nathalie Scheers

**Affiliations:** 1 Department of Life Sciences, Chalmers University of Technology, 412 96 Gothenburg, Sweden; 2 Department of Laboratory Medicine, Lund University, 221 00 Lund, Sweden; 3 Department of Veterinary Medicine, University of Cambridge, Cambridge CB3 0ES, UK

**Keywords:** iron, ferric pyrophosphate, amphiregulin, COX-2, IFNGR1, IGF1R, IL-6

## Abstract

We previously showed that two iron compounds that are orally ingested by humans, namely ferric EDTA and ferric citrate, can induce an oncogenic growth factor (amphiregulin) in human intestinal epithelial adenocarcinoma cell lines. Here, we further screened these iron compounds, plus four other iron chelates and six iron salts (i.e., 12 oral iron compounds in total), for their effects on biomarkers of cancer and inflammation. Ferric pyrophosphate and ferric EDTA were the main inducers of amphiregulin and its receptor monomer, IGFr1. Moreover, at the maximum iron concentrations investigated (500 µM), the highest levels of amphiregulin were induced by the six iron chelates, while four of these also increased IGfr1. In addition, we observed that ferric pyrophosphate promoted signaling via the JAK/STAT pathway by up-regulating the cytokine receptor subunit IFN-γr1 and IL-6. For pro-inflammatory cyclooxygenase-2 (COX-2), ferric pyrophosphate but not ferric EDTA elevated intracellular levels. This, however, did not drive the other biomarkers based on COX-2 inhibition studies and was probably downstream of IL-6. We conclude that of all oral iron compounds, *iron chelates* may particularly elevate intracellular amphiregulin. Ferric pyrophosphate additionally induced COX-2, probably because of the high IL-6 induction that was observed with this compound.

## 1. Introduction

Dietary iron has been associated with several cancer types, including colorectal cancers. In the published literature, a distinction between *iron exposure* and body *iron stores* in relation to cancer risk can be made. A systematic review and meta-analysis of epidemiological studies between 1995 and 2012 [1] indicated that intake of dietary iron and, in particular, heme-iron (an iron chelate) was associated with colorectal and colon cancer risk if heme-iron intake was >1 mg/day (colorectal: relative risk (RR): 1.08; 95% CI: 1.00–1.17 and for colon: RR = 1.12; 95% CI, 1.03–1.22). The same study observed that high levels of biomarkers for iron stores were associated with *decreased* cancer risk. Examining an earlier review from 2001 [2], in which 26 papers from 1985 and onwards were included, the same distinction can be made when iron exposure and body iron store outcomes are separated. We interpret these differences as meaning that some iron from dietary exposure does not end up in body stores but is still potentially available to promote cancerous growth and pro-inflammatory characteristics as it transits the colon.

In keeping with the above, mechanistic work has shown that iron promotes cell growth [3,4], and, for the intestine, this appears to be related to the initial iron formulation [5] and thus the nature of interaction with the absorptive cells of the gut mucosa. Moreover, in two murine colitis-associated carcinogenesis models (DSS-induced colitis and interleukin 2 knockout), tumor burden in response to dietary iron was observed to increase in comparison to parenteral iron administration [6,7]. In addition, in a long-term oral iron administration study in the DSS-induced colitis model, dietary iron increased tumor incidence, while parenteral iron did not [8]. In these studies, the ‘dietary’ iron was supplemented into the standard rodent chow diet and was ferric EDTA with, on one occasion, ferric citrate in addition (both are iron chelates). Indeed, our earlier cell work [5] showed that human intestinal epithelial adenocarcinoma cells (Caco-2 and Hutu-80), treated with ferric EDTA or ferric citrate, presented with elevated levels of the growth factor and tumor marker amphiregulin (also referred to as AREG) in addition to one of its receptors, EGFr, followed by activation of the oncogenic MAP kinase pathway by means of downstream phosphorylation of ERK. In the control cells, untreated or treated with ferrous sulfate, there was no activation of the MAP kinase pathway.

Amphiregulin has been observed to be crucial in several steps of tumorigenesis, such as self-sufficiency in inducing growth signaling, replicative ability, metastasis, angiogenesis, and blocking apoptosis, as investigated in human cell lines and rodents, reviewed in [9]. The involvement of amphiregulin in oncogenic progression in humans has been observed in common cancers, including lung [10], breast [11], ovarian [12], and colorectal cancers. So germane is the role of amphiregulin that it has been proposed as a prognostic marker in malignant colorectal cancers [13], and it is associated with chemotherapy resistance [14,15]. Accordingly, cancer drugs targeting amphiregulin RNA expression are being developed [16]. Another growth-promoting phosphorylation cascade, namely the JAK/STAT pathway that is similarly activated by binding of substrates to receptors such as EGFr, has been observed to be involved in colorectal cancer progression [17]. The JAK/STAT pathway is also engaged in immune responsiveness, and the hallmark of ‘flow’ through this pathway is the cytokine IL-6, connecting tumor growth to inflammation.

To find a plausible mechanism for amphiregulin induction and to investigate the dependence on initial iron formulation, we investigated the inducible cyclooxygenase (COX-2) pathway, which produces inflammatory and pain-stimulatory mediators and, especially, stimulates Prostaglandin E2 (PGE2) production. PGE2 promotes amphiregulin expression and plays an essential role in colorectal cancer progression [18,19]. Interestingly, in rabbits, PGE2 levels have been shown to be elevated by iron chelate, ferric nitrilotriacetate (FeNTA) [20]. In the present work, we chose to investigate 12 different iron compounds that are used for dietary supplementation or fortification of foods. These compounds contain high- and low-affinity chelates of ferric (Fe^3+^) and ferrous (Fe^2+^) iron or are iron salts. As such, we investigated associations between the amphiregulin stimulating effect and the chemical form of the iron compound (chelates vs. salts). We also extended biomarkers for cancer/inflammation (i.e., beyond just amphiregulin) with a proteomic approach using targeted human proteomic panels (OLink proteomics, Uppsala, Sweden). Finally, we commenced work on the involvement of the COX-2 pathway in the induction of amphiregulin.

## 2. Results

### 2.1. Ferric Pyrophosphate—A Major Novel Inducer of Amphiregulin

**Figure 1:**
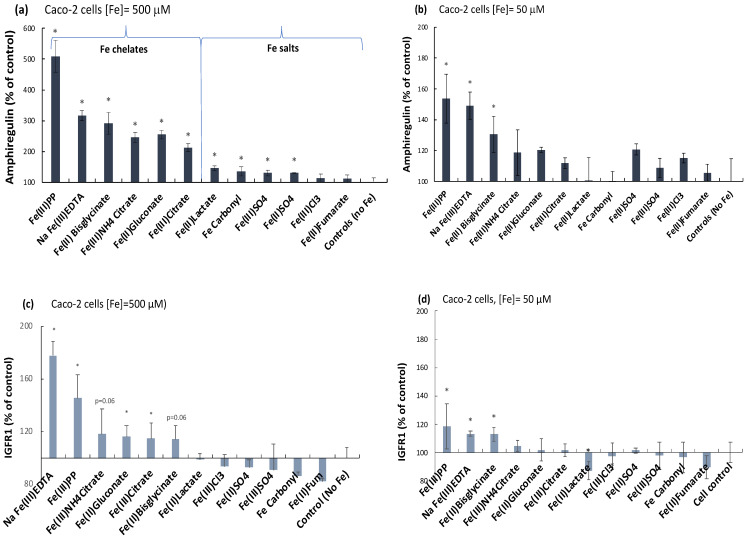
Amphiregulin (**a**,**b**) and IGFr1 (**c**,**d**) levels (% of untreated controls) in intestinal Caco-2 cells treated with iron compounds (500 µM and 50 µM, 48 h). Relative intracellular levels were analyzed with proteomic profiling (Olink^®^ Target 96; Oncology II) OLink proteomics, Uppsala, Sweden. A significant difference from untreated control cells is indicated with an asterisk (*). For Caco-2 cell viability data (cell survival), please refer to Figure S1a).

Out of 12 iron compounds tested at 500 µM Fe (translating to a 170 mg oral dose in humans [21]), the 6 iron chelates all elevated amphiregulin levels, whereas only 4 of the iron salts caused such an effect and at lower levels than for the chelates (Figure 1a). For example, the increase in amphiregulin in cells treated with ferric citrate (i.e., the chelate with the least effect) was still greater than the amphiregulin increase from the cells treated with the iron salt with the greatest effect (ferrous lactate, a monodentate ligand, defined as salt; *p* = 0.001). Among the iron chelates, amphiregulin production was observed to be more extensive after cell treatment with ferric pyrophosphate than with ferric EDTA (*p* = 0.004; Figure 1a). However, with a ten-fold lower iron load of 50 µM, there was no significant difference between these two high-affinity ferric chelates (*p* = 0.66; Figure 1b).

### 2.2. Elevation of an Amphiregulin Receptor Monomer, IGFr1

**Figure 2:**
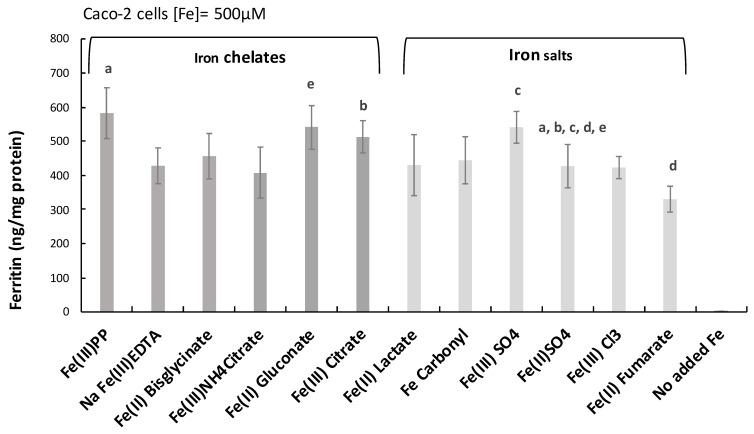
Iron load in response to different compounds was compared to ‘the gold standard’ ferrous sulfate. Intracellular ferritin levels indicate iron load (cellular stores). Data are normalized to total cellular protein and are means of 6 cell replicates ± Sdev (n = 2). The baseline ferritin level in control cells (no added iron) was 1.3 ± 0.8 ng/mg protein. A copy of the same letter indicates a significant difference (*p* < 0.05).

The monomer IGFr1 of the IGF receptor, for which amphiregulin is a substrate in addition to the EGF receptor, was also elevated by the iron chelates at 500 µM (Figure 1c), albeit less extensively in comparison to the substrate amphiregulin itself. Any difference between ferric pyrophosphate and ferric EDTA treatments was borderline (*p* = 0.06). At the lower exposure (50 µM, 48 h), only ferric pyrophosphate, ferric EDTA, and ferrous bisglycinate induced IGFr1 significantly (*p* = 0.05, *p* = 0.000004, and *p* = 0.04, respectively; Figure 1d). There was no significant effect on IGFr1 in the presence of iron salts. Overall, the results suggest that the iron-stimulatory effect on the MAP kinase pathway mediators amphiregulin and IGFr1 are almost exclusively associated with the chelating ability of the iron ligand (Figure 1a–d) and, generally, was greatest for the ligands pyrophosphate and EDTA. Importantly, the trend for the two types of iron compounds (iron chelates vs. iron salts) did not correlate with absorbed and stored cellular iron (ferritin levels), indicating that the effect was not caused by higher iron uptake from the iron chelates than iron salts, neither was the trend associated with iron oxidation state (ferric: III or ferrous: II) (Figure 2).

### 2.3. Induction of the Inflammatory Mediators IFN-γ r1, CDKNA, and IL-6 by Ferric Pyrophosphate: Engagement of the JAK/STAT Pathway

**Figure 3:**
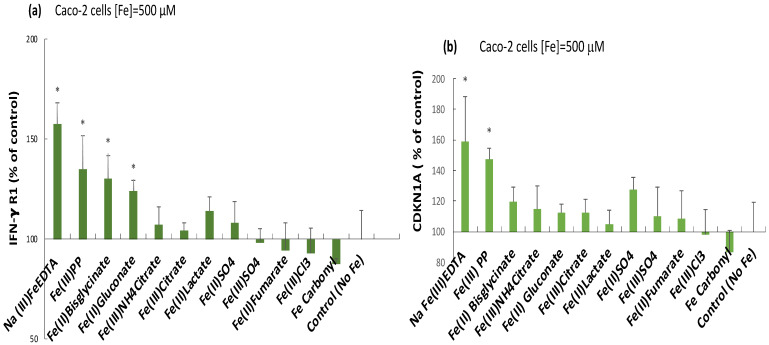
Relative intracellular levels of (**a**), the interferon γ receptor monomer IFN-γ r1 (**b**), the ERK up-regulator CDKN1A in response to iron compounds (500 μM, 48 h). Linearized, normalized protein expression data from iron-treated cells as a percentage of untreated controls. Method: Olink proteomics. Data are means of 3 cell replicates ± Sdev. An asterisk (*) indicate a significant difference from control cells, *p* < 0.05.

**Figure 4:**
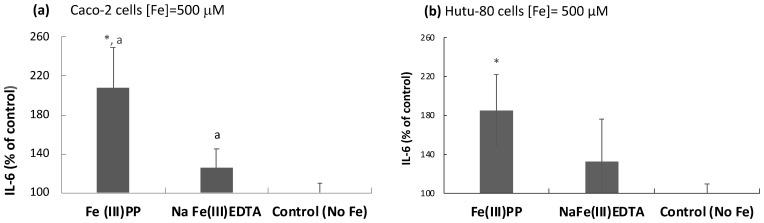
Intracellular IL-6 production in response to ferric pyrophosphate. (**a**) In Caco-2 cells; linearized, normalized protein expression of IL-6 presented as a percentage of untreated controls. Method: Olink proteomics. (**b**) In Hutu-80 cells; measured with a commercial ELISA kit (#DY206, R&D Systems). Data are means of 3 cell replicates ± Sdev. The asterisk indicates a significant difference from untreated control cells, *p* = 0.01 in (**a**) and *p* = 0.02 in (**b**). A significant difference between the iron treatments is indicated by the letter a (*p* = 0.04). Significant differences from the control (no Fe) are indicated with asterisks (*).

The cytokine receptor subunit IFN-γ r1 levels were significantly elevated in response to all iron chelates, except for ferric citrate, at 500 µM Fe (Figure 3a). There was no difference (*p* = 0.13) between ferric pyrophosphate and ferric EDTA treatments, and they induced the highest changes overall. At lower iron exposure (50 µM), there were no significant changes from untreated control cells (*p* > 0.05, data not shown). That IFN-γ r1 monomer levels showed a similar trend to amphiregulin and the IGF r1 monomer suggests a connection of the induction of the MAP kinase pathway with signaling via the JAK/STAT pathway in response to (especially) ferric pyrophosphate (Figure 3a), which was further evidenced by the production of the pro-inflammatory cytokine IL-6 and its target CDKN1A (Figure 3b). CDKNA was elevated by ferric pyrophosphate (*p* = 0.006) and ferric EDTA (*p* = 0.007) at the higher iron exposure (500 µM) (Figure 3b). Similar to IFN-γ r1, the lower level (50 µM, 48 h) did not induce CDKN1A expression (*p* > 0.05, data not shown). CDKN1A is an up-regulator of ERK and may therefore potentiate the MAP kinase pathway by increasing available ERK (inactive) as a positive feedback mechanism. Although IL-6 has been described to be an inducer of the pro-inflammatory CDKN1A [22], ferric EDTA (at 500 µM, 48 h) did not significantly increase the production of IL-6 (*p* = 0.1) in contrast to ferric pyrophosphate (Figure 4).

### 2.4. Ferric Pyrophosphate, but Not Ferric EDTA, Significantly Elevated Intracellular COX-2

**Figure 5:**
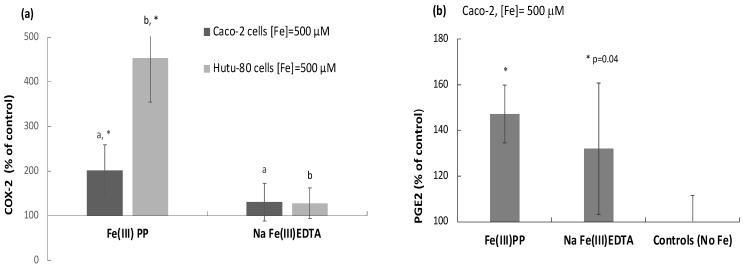
(**a**) COX-2 levels (% of control) in Caco-2 and Hutu-80 cells measured with ELISA (#DYC4198-2 R&D systems). Data are presented as means of 7 cell replicates ± Sdev from 2 separate occasions (triplicate + quadruplicate). A copy of the same letter indicates significant differences between the iron treatments (a or b). (**b**) The COX-2 product PGE2 intracellular levels (% of control) in Caco-2 cells were measured with ELISA. Data are presented as means of 6 cell replicates ± Sdev from 2 separate occasions. Significant differences from the control (no Fe) are indicated with asterisks (*).

To further investigate the stimulatory effect of the highly active chelates (ferric pyrophosphate and ferric EDTA) on the Map kinase pathway and JAK/STAT mediators, we focused on the inducible cyclooxygenase (COX-2) pathway as outlined in the Introduction. In the two human intestinal cell lines studied, ferric pyrophosphate (500 μM) significantly elevated COX-2 production in comparison to control (Caco-2: *p* = 0.001, Hutu-80: *p* = 0.000003) or ferric EDTA (Caco-2: *p* = 0.02, Hutu-80: *p* = 0.000002) (Figure 5a). The COX-2 product, prostaglandin E2 (PGE2), was also measured in the Caco-2 cells. Consistently, ferric pyrophosphate significantly increased intracellular PGE2 levels (*p* = 0.00005), while ferric EDTA caused a variable but just-significant increase (*p* = 0.04, Figure 5b).

### 2.5. COX-2 Appears to Be Downstream of IL-6 as Indicated by COX-2 Inhibition Studies

**Figure 6:**
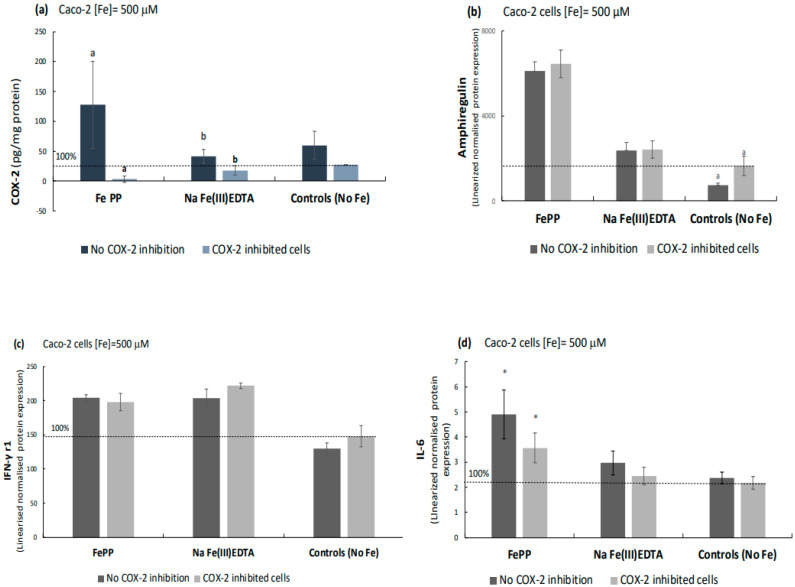
COX-2 inhibition in Caco-2 cells by the selective COX-2 inhibitor sulindac sulfone. (**a**) COX-2 levels (pg/mg protein) in iron-treated Caco-2 cells, with and without COX-2 inhibition, measured with a commercial ELISA kit (#DYC4198-2 R&D systems)**.** Data are means of 6 cell replicates ± Sdev (**b**) amphiregulin (**c**) IFN-γ r1 (**d**) IL-6 levels are expressed as linearized normalized protein expression. Data are means of 3 cell replicates ± Sdev. Significant differences between treatments are indicated with a copy of the same letter, while significant differences from control (no iron treatment) are indicated with asterisks (*). For Caco-2 cell viability data (cell survival), please refer to Figure S1b).

To establish if COX-2 drives amphiregulin or IL-6 production in the iron-treated intestinal cells, the selective COX-2 inhibitor sulindac sulfone (SS) was used. The results reveal that COX-2 in SS/iron-treated cells was significantly inhibited ([SS] = 100 μM, 72 h) in comparison to its non-inhibited control (Fe(III)PP: 98%, *p* = 0.04 and Na Fe(III)EDTA: 57%, *p* = 0.04; Figure 6a). Amphiregulin and IFN-γ r1 levels in iron-treated cells were not significantly affected by the COX-2 inhibition (Figure 6b,c). There was a small but insignificant difference in IL-6 levels in COX-2 inhibited vs. non-inhibited ferric pyrophosphate-treated cells (*p* = 0.11, Figure 6d). However, the significant increase in cellular IL-6 levels in the presence of ferric pyrophosphate in COX-2-inhibited cells compared to COX-2-inhibited control cells (with no additional iron, *p* = 0.02) indicates that COX-2 is downstream to IL-6 (may be specific to intestinal cells). In addition, that IL-6 drives COX-2, and not the other way around, explains why ferric EDTA did not significantly elevate COX-2 protein levels in either Caco-2 or Hutu-80 cells (Figure 5a) during the present conditions. The elevation of amphiregulin, IFN-γ r1, and IL-6 observed for the non-COX-2 inhibited cells in the COX-2 inhibition studies were consistent with the findings reported in Figure 2, Figure 3 and Figure 4 (amphiregulin, IFN-γ r1, and IL-6).

## 3. Discussion

### 3.1. Rationale for Investigating Effects of Iron Compounds in Human Adenocarcinoma Cells

The association between enhanced tumorigeneses of the colon and iron exposure has been demonstrated over several studies and a number of years. Our work has indicated that different forms of iron induce different cellular effects, and we, therefore, seek to identify potential mechanisms for these differential effects in this work and elsewhere [5] to understand and possibly answer questions such as if some iron forms are potentially more tumorigenic than others. This is especially important today when so many different forms of iron are available as fortificants, supplements, and even phosphate binders in renal patients. Continuing such investigations should help identify if these effects carry through to humans and if they can be obviated through the choice of oral iron compound.

### 3.2. The Effect of Iron Chelates and Iron Salts on Growth/Inflammatory Pathways

The engagement of the MAP kinase pathway, by means of elevated amphiregulin levels, was evident for all the investigated iron chelates, while the increase in the amphiregulin receptor monomer IGFr1 was a little less pronounced, i.e., the percentage increase in amphiregulin was larger as compared to the percentage increase in IGFr1. Noteworthy is that in the case of amphiregulin, the levels in the cells were increased from the untreated control cells also in the presence of the higher dose of the iron salts (500 μM), though with a significantly lower effect than the iron chelates. This was the only case in which the iron salts had a significant effect on the investigated biomarkers. It was also observed that ferric pyrophosphate and ferric EDTA affected the MAP kinase pathway mediators more than the other chelates, and it was established that this effect was not due to an increased cellular iron load. Our previously published data showing significant effects on amphuregulin elevation by ferric EDTA and ferric citrate, but not ferrous sulfate, observed with two different methods (proteome profiler^TM^ arrays and ELISA) confirm these findings using the Olink proteomic approach. Albeit, in some cases, the *absolute* changes vary to some extent between the studies using the different methods, which, however, are to be anticipated, and the important aspect is that the overall enhancing or non-enhancing effect (rather than the precise magnitude) is the same.

In addition, the engagement of the JAK/STAT pathway through elevation of the interferon gamma receptor subunit IFN-γ r1 and the multifunctional cyclin-dependent kinase inhibitor CDKNA by these two iron chelates, independent of dose (50 or 500 μM) was also evident. In addition to stimulating signaling via these two pathways associated with growth, proliferation, and angiogenesis, ferric pyrophosphate treatment also induced the cytokine IL-6, which, in turn, engaged the inducible COX-2 pathway of inflammation. Ferric EDTA treatment did not come out as a significant stimulant of IL-6 at the highest concentration (500 μM), and thereby, COX-2 was not induced. The significant increase in PGE2 in the presence of ferric EDTA, although with a large variance between experiments, could be a result of the COX-1 enzyme, which is constitutively expressed in most tissues but not induced by inflammatory mediators (such as IL-6). Speculatively, iron compounds, as such, could induce a baseline level of PGE2 through COX-1, but only COX-2, as induced by IL-6, would further elevate PGE2.

### 3.3. Iron Exposure and Load

Here we investigated the influence of dietary iron supplementation and food fortification compounds at levels relevant to intestinal levels of available iron after exposure. According to a study by Glover and Jacobs [19] supported by [23], the postprandial ionizable iron concentration in the intestinal lumen after a standard meal containing ~62 µmol (3.45 mg) iron was reported as 10 µM (i.e., a 6.2-fold dilution), which would imply that in the present study, the single doses of given iron (50 µM and 500 µM) translate into an iron exposure of approximately 310 µmol (~17 mg) and 3.1 mmol (~170 mg) per dose. In a study by Lund et al. [24] in 18 healthy volunteers, the baseline iron content in stool was 60 µM, and after 2 weeks of supplementation with 100 mg ferrous sulfate per day, the stool iron was increased to 300 µM, supporting the assumptions above. Standard iron supplementation doses are in the range of 20 to 200 mg per 24 h depending on the severity of the iron deficiency and can be even higher (>600 mg/10.7 mmol of luminal iron) in the case of phosphate binders (e.g., ferric citrate) used in renal disease therapies [25]. Our results show that there is a dose dependence (in relation to iron exposure) of the cellular response on the investigated biomarkers. In the present study, we used ferritin as a proxy for iron load, as it has been established that the cellular iron storage protein ferritin is proportional to iron uptake in the Caco-2 cell model [26]. Consistent with the findings in humans (described in the Introduction), iron load, as stored iron, was not associated with increased biomarkers for cancer. However, with iron exposure and mainly from the iron chelates, such associations were found. It is intriguing that intake of heme-iron, another iron chelate, is associated with colon cancer risk since it, too, is a strong coordination complex (chelate) of iron.

## 4. Materials and Methods

### 4.1. Iron Compounds

Ferric pyrophosphate (soluble crystals), ferric sodium EDTA hydrate, ferric ammonium citrate, ferric citrate hydrate, ferrous gluconate hydrate, carbonyl iron, ferrous lactate hydrate, ferric chloride hexahydrate, ferrous fumarate, ferrous sulfate heptahydrate, and ferric sulfate hydrate were purchased from Sigma-Aldrich (St. Louis, MO, USA). Ferrous bisglycinate was purchased as a dietary supplement in capsules targeted for consumers from the online store Supersmart (headquarters in Luxembourg). Stock solutions ([Fe] = 5 mM) of each compound were made fresh before each experiment.

In the initial experiments, ferrous fumarate, ferrous bisglycinate, ferric pyrophosphate, and carbonyl iron were dissolved in hydrochloric acid (50 mM), while the others were dissolved in ultrapure water. In later experiments, dissolution in hydrochloric acid was omitted.

### 4.2. Microwave Digestion and Atomic Absorption Spectroscopy (AAS)

The iron content of the stock solutions was verified by AAS. Briefly, each stock solution (1 mL) was mixed with ultrapure water (3–4 mL), concentrated nitric acid (0.75 mL; trace metal grade; Fisher Scientific, Loughborrough, U.K.), and concentrated hydrochloric acid (0.15 mL; trace metal grade; Fisher Scientific, Loughborrough, U.K.) in Teflon microwave digestion vessels. The microwave digestion was then carried out in an Ethos plus microwave lab station (Milestone; Sorisole, Italy) at 180 °C for 15 min. Iron concentration in the microwave-digested samples was determined using an Agilent 240/280 Series AA spectrometer (Santa Clara, CA, USA).

### 4.3. Cell Culture

The colorectal adenocarcinoma [Caco-2 (ATCC^®^ HTB37)] was mainly used, and the duodenal adenocarcinoma [Hutu-80 (ATCC^®^ HTB40)] was used to exclude cell line-specific effects in the COX-2 studies (We have previously shown data on MAP kinase pathway activation in both Caco-2 and Hutu-80 cells [5]). Both cell lines were cultured in minimum essential medium (MEM; Gibco, Thermo Fisher Scientific, Rockford, IL, USA) supplemented with heat-inactivated fetal bovine serum (FBS; 10%; Gibco), L-glutamine (2 mM; Gibco), and normocin (0.1 mg/mL; Invivogen, Toulouse, France) at 37 °C and 5% CO_2_ with medium change every two to three days. The cells were passaged at reaching 80% confluency using trypsin-EDTA (0.05%; Gibco).

### 4.4. Cell Experiments

Caco-2 (p. 24–36) and Hutu-80 cells (p. not retrievable) were seeded in 12- or 24-well plates (Corning Life Sciences, Lowell, MA, USA), at a density of 200,000 or 100,000 cells/well. The medium (MEM 5% FBS, 0.2% normocin) was supplemented with iron solutions, except for controls, at [Fe] = 50 µM and 500 µM. The supplemented medium was aspirated after 48 h incubation, and the cells were washed with DPBS (Dulbecco’s phosphate-buffered saline without calcium and magnesium; GE Healthcare Life Sciences, Logan, UT, USA) and lysed in cold RIPA buffer (200 µL for 12-well plates and 100 µL for 24-well plates; Sigma-Aldrich, St. Louis, MO, USA) with added protease inhibitors (Thermo Fisher Scientific, Rockford, IL, USA). The lysates were collected and frozen at −80 °C until analysis. Aliquots of cell lysates were analyzed for total protein (BCA assay, Pierce, Chicago, IL, USA) and ferritin (# EIA1872, DRG, Springfield CA, USA) according to the manufacturer’s protocols. Ferritin data were normalized to the total cellular protein from each well.

### 4.5. COX-2 Inhibition Studies

Caco-2 cells were seeded and cultured as described above. The experimental setup was that seeded cells were left to grow in the incubator for 24 h prior to incubating the cells with the selective COX-2 inhibitor sulindac sulfone, 100 µM per well in DMSO (0.1% final concentration). The incubation lasted for 24 h, and then cells were given fresh MEM (FBS 5%) supplemented with sulindac sulfone and iron compounds ([Fe] = 500 µM). After 48 h, the cells were washed and lysed in cold RIPA (Sigma-Aldrich, St. Louis, MO, USA) supplemented with protease inhibitors (Thermo Fisher Scientific, Rockford, IL, USA). Control experiments with the same setup but without the sulindac sulfone addition were carried out in parallel. Cell survival was estimated by means of total protein (BCA assay, Pierce, Chicago, IL, USA) remaining after washing the cells at the end of the experiments.

### 4.6. Biomarkers for Inflammation

Intracellular levels of IL-6 (#BMS213-2, Thermo Fisher Scientific, Rockford, IL, USA) and COX-2 (#DYC4198-2 R&D systems) were measured by ELISA according to the manufacturer’s instructions. ELISA data were normalized to the total cellular protein from each well.

### 4.7. Proteomic Analysis of Biomarkers for Inflammation and Cancer

Relative changes in cellular levels of amphiregulin, IGFr1, IFN-γ r1, CDKNA, and IL-6 in cells treated with iron, as compared to untreated control cells, were identified by the OLink target 96 oncology II panels run by OLink Proteomics Uppsala, Sweden. Briefly, cell lysates were sent to Olink at a protein concentration of 0.25 or 0.5 μg/μL in 96-well plates. The analysis is carried out in the same plate using Olink proximity extension assay (PEA) technology in which 92 oligonucleotide-labeled antibody probe pairs are allowed to bind to their target proteins in the sample. This binding forms a PCR reporter sequence by proximity-dependent DNA polymerization. The reporter sequences are subsequently amplified and detected using real-time PCR [27].

### 4.8. Statistics

Student’s unpaired *t*-test (Microsoft^®^ Excel, Redmond, WA, USA) was used to estimate the significance of differences. Two tails and equal variance or unequal variance were used where appropriate (2,2) or (2,3). Data are means of at least 3 cell replicates ± Sdev. Some experiments are reported separately as n = 1 (with 3 or 4 cell replicates) since they were part of different trials; however, all experiments were repeated at least once (n = 2).

## 5. Conclusions

All six investigated iron chelates, common as dietary supplements or food fortificants, elevated the pro-cancerous biomarker amphiregulin in the human intestinal adenocarcinoma cell line (Caco-2) at intestinal-relevant levels. As demonstrated with ferric pyrophosphate and ferric EDTA, the pro-inflammatory enzyme COX-2 was not a direct mediator in amphiregulin induction but was induced by the inflammatory cytokine IL-6, which in turn was induced by ferric pyrophosphate under the present conditions. These pro-cancerous and pro-inflammatory effects were related to the level and type (iron form) of cellular exposure but were not associated with iron load (ferritin storage). These in vitro data require further investigation and translation to in vivo studies (limitations of cell models are reviewed in [28]) to evaluate whether iron chelates should be avoided in iron supplementation and food fortification.

## Data Availability

All data are available from the corresponding author upon reasonable request.

## Supplementary Materials

The following supporting information can be downloaded at: https://www.mdpi.com/article/10.3390/ijms24065507/s1.
