# Supplementary material for: Differential Effects of Iron Chelates vs. Iron Salts on Induction of Pro-Oncogenic Amphiregulin and Pro-Inflammatory COX-2 in Human Intestinal Adenocarcinoma Cell Lines
Source: Int J Mol Sci. 2023 Mar 14;24(6):5507. doi: 10.3390/ijms24065507 (PMC10051564; doi:10.3390/ijms24065507)
Supplement: Supplementary file 1 [file ijms-24-05507-s001.zip › ijms-2225468-supplementary.pdf]

Supplementary Figure S1.

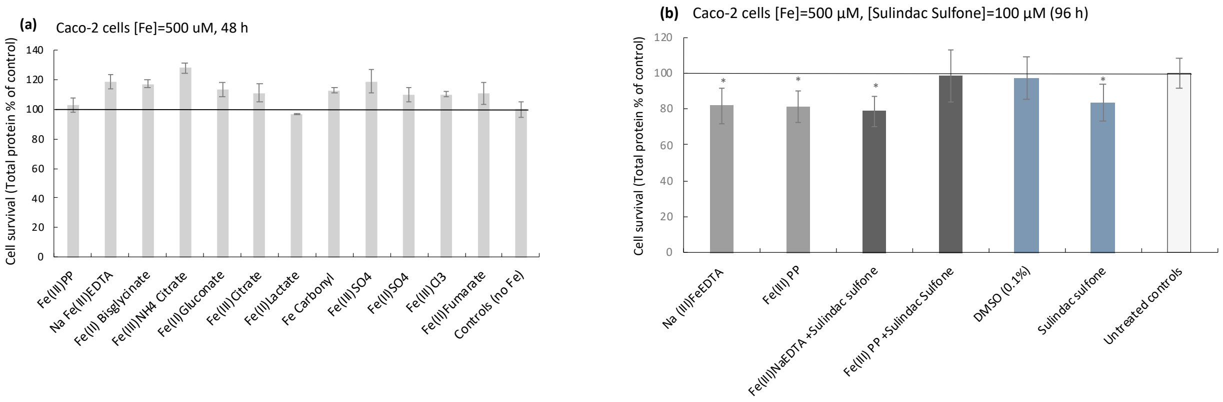

Figure S1. Cellular viability data (cell survival) for **a**) experiments with iron compounds at 500 µM, 48 hours **b)** COX-2 inhibited cells (Sulindac sulfone at 100 µM, 96 hours). Data are means of 3 cell replicates ± Sdev. Significant differences from untreated control cells are indicated with an asterisk (*).
